# A Diagnostic System for Improving Biomass Quality Based on a Sensor Network

**DOI:** 10.3390/s110504990

**Published:** 2011-05-04

**Authors:** Dionysis D. Bochtis, Claus G. Sørensen, Ole Green, Thomas Bartzanas

**Affiliations:** 1 Department of Biosystems Engineering, Aarhus University, Blichers Allé 20, 8830 Tjele, Denmark; E-Mails: claus.soerensen@agrsci.dk (C.G.S.); ole.green@agrsci.dk (O.G.); 2 Center for Research and Technology of Thessaly, Institute of Technology and Management of Agricultural Ecosystems, 38333 Volos, Greece; E-Mail: thomas.bartzanas@gmail.com

**Keywords:** decision support system, computational fluid dynamics, biomass

## Abstract

Losses during storage of biomass are the main parameter that defines the profitability of using preserved biomass as feed for animal husbandry. In order to minimize storage losses, potential changes in specific physicochemical properties must be identified to subsequently act as indicators of silage decomposition and form the basis for preventive measures. This study presents a framework for a diagnostic system capable of detecting potential changes in specific physicochemical properties, *i.e.*, temperature and the oxygen content, during the biomass storage process. The diagnostic system comprises a monitoring tool based on a wireless sensors network and a prediction tool based on a validated computation fluid dynamics model. It is shown that the system can provide the manager (end-user) with continuously updated information about specific biomass quality parameters. The system encompasses graphical visualization of the information to the end-user as a first step and, as a second step, the system identifies alerts depicting real differences between actual and predicted values of the monitored properties. The perspective is that this diagnostic system will provide managers with a solid basis for necessary preventive measures.

## 1. Introduction

Silage losses are the main parameter that defines the profitability of the silage production and affects the animals in terms of nutrition and hygienic conditions. In order to preserve the nutritional quality of stored biomass, certain essential conditions need to be met during the storage process (e.g., [1]). Insufficiently maintained cover systems (e.g., tears in the plastic covering, cracks in the walls) cause rapid decomposition of the adjacent silage resulting in the dry matter being broken down into H_2_O and CO_2_ with a subsequent release of heat [2].

In order to ensure adequate preservation of the silage during the entire storage period, it is important to be able to detect potential changes in specific physicochemical properties, such as the temperature and the oxygen content of the silage, that can act as indicators of silage decomposition and form the basis for preventive measures. It has been shown that it is not possible to detect visibly the silage decomposition under the sealed cover leading to unavoidable dry mass losses that can reach levels as high as 75% of the total silage before the actual decomposition is even remotely visible [3]. Consequently, there is perceived need for the development and implementation of dedicated decision systems for the prediction of quality parameters in stored biomasses, for example, by predicting the occurrence of oxygen entering the stack by monitoring the temperature and the outside weather conditions and incorporating such measures into designated decision support systems and by extension into farm management information systems [4,5]. Such decision support systems will enable an early and timely detection of process disturbances, which subsequently can be evaluated and provide the guidelines for possible preventive measures. This will enable the manager of silage storage facilities to accurately monitor and prognosticate vital quality parameters of the silage as part of day to-day planning and control of the storage conditions on a continuous basis.

Traditional invasive monitoring systems to evaluate the condition of the silage have been used (e.g., [6,7]). However, these monitoring systems themselves have a negative impact on the preservation of the silage stack since the measurement processes are destructive to the airtight sealing of the silage stack, causing silage to come into contact with O_2_ and resulting in decomposition of its digestible matter. In any case, non-invasive novel monitoring systems, such as wireless sensors capable of precisely measuring silage quality parameters, are preferable. Recently, Green *et al*. [8] presented and evaluated a system composed of novel non-invasive wireless nodes capable of measuring the temperature and oxygen inside silage stacks. The developed monitoring system was found to be highly accurate, indicating that the designed wireless sensor nodes could potentially be used for detecting the occurrence of silage decomposition.

A fully operational decision support will require the comparison or benchmarking between the monitored and the preferred conditions. Consequently, any sensor network system should be combined with a model for the prediction of the physicochemical properties that are being monitored. A number of analytical models have been developed to quantify the deterioration of silage over time (e.g., [9,10]). Nevertheless, these models do not provide any information concerning the distribution of the physico-chemical parameters inside biomass storage facilities. In order to provide the lacking distribution in time and space of these parameters numerical modelling approaches seem to be promising as it has been proven for developed computational fluid dynamics models for biomass conventional storage systems such as for forage crops [11] or grains [12,13].

Wireless sensor networks provide low-cost and low-power deployments in a number of agricultural-related applications [14,15], including, for example, irrigation [16–19], environmental monitoring [20], specialty crops [21,22], viticulture [23], and animal production [24,25]. In this paper, a diagnostic system comprising both monitoring by a wireless sensor network, and prediction by a computational fluid dynamics model, of governing biomass quality parameters will be developed and will form the basis for any preventive measures to be implemented by the manager.

## 2. System Overview

The proposed diagnostic tool involves a combination of a network of sensors for the acquisition of real-time data in order to provide information about actual conditions within the biomass storage facility and a real-time computational fluid dynamics (CFD) modelling approach fitted with selected updated weather data in order to provide the predicted conditions within the storage facility assuming ideal conditions with no interfering events. The two conditions (actual and predicted) are depicted graphically in a user interface allowing the end user to evaluate any deviation between the two conditions and decide if preventive actions are required. In more detail, the principal components of the diagnostic system include:

– A sensor network functioning as a data acquisition unit. The measured data include the air temperature and the oxygen content within the biomass storage volume.
– A wireless communication unit for the transmission of sensor data to an on-site data storage unit.
– The data storage unit. This unit serves as an intermediate receiver of acquired data which subsequently transmit this data to a dedicated user interface through an internet connection.
– A central server running the CFD model. This modelling unit uses as input the current weather conditions and simulates the course of the air temperature and the oxygen content in time and space. The simulation assumes a geometrical configuration similar to the actual one and appropriate physical properties of the material.
– A server providing updated weather data required by the CFD model in a fixed time sequence. For the specific model, the required weather data include the (outside) air temperature, relative humidity, wind speed and direction, global solar radiation, and surface and soil temperatures.
– A user interface for visualization purposes. It receives in a regular time-base the two sets of conditions and provides a graphical direct comparison.

**Figure 1.**
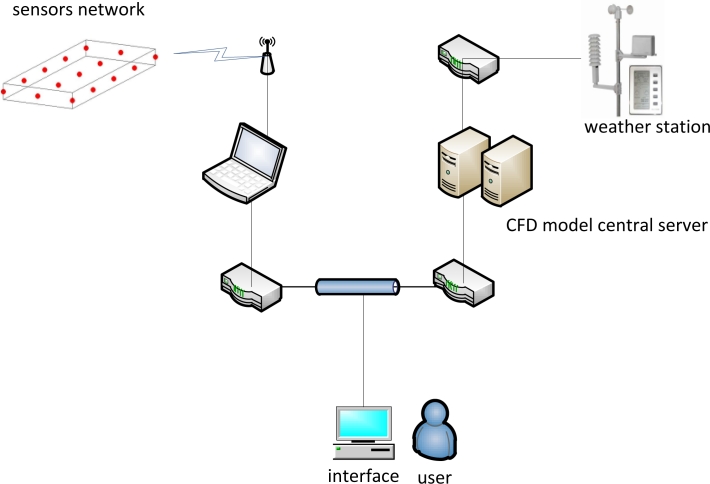
The basic physical components of the diagnostic system.

These parts are depicted in Figure 1. The following sections detail the individual components of the above described system. Specifically, in the next section the sensor unit employed is described. Following this section, the CFD model is described in terms of mathematical formulation, biomass modelling approach, specification of the boundary conditions, and finally the validation of the model. Next, in order to demonstrate the functionality of the system a specific scenario based on experimental data is indentified.

## 3. The Sensor Network

**Figure 2.**
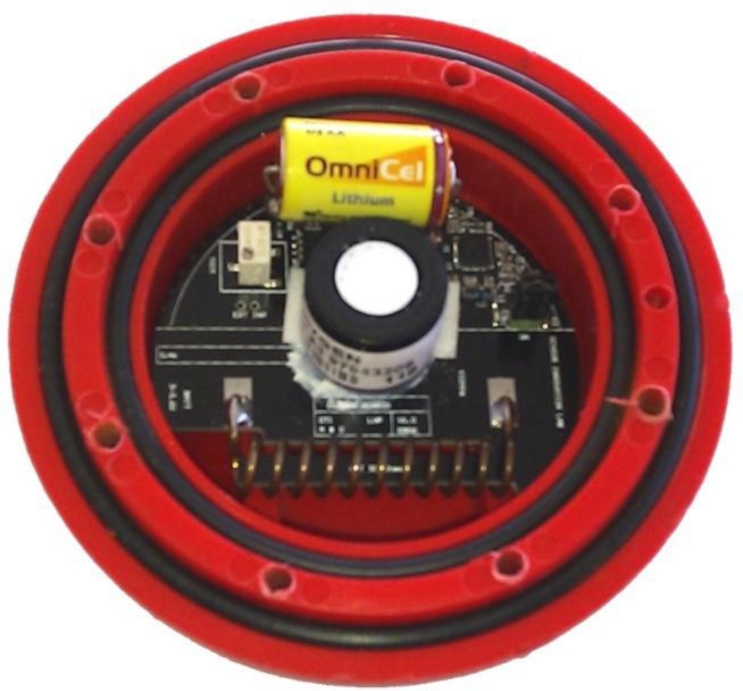
The sensor unit for temperature and oxygen consecration measurements within the protective housing.

The sensor network combines a number of specific sensor units for measuring temperature and oxygen concentration designed by Green *et al.* (Figure 2) [8]. The sensor unit is a single-chip system with fully integrated (433 MHz) RF transceiver, 8051-compatible microcontroller and a four-input, 10-bit, 80 ksps A/D converter. The circuit has embedded voltage regulators, which provides maximum signal to noise immunity and allows operating on a single 1.9–3.6 V supply. The transceiver consists of a fully integrated frequency synthesizer, a power amplifier, a modulator, and a receiver unit. Output power and frequency channels and other RF parameters are programmable by use of the on-chip serial programmable interface to the sensor core. Each sensor node acts as a transmit-only device in a single-hop broadcast network and the data are received by a gateway node. Each sensor node actively participates in handshaking communication. Therefore, acknowledgment messages are sent back to the originating node when the sensor messages are received by the gateway. The acknowledgment messages might include information relevant for network re-tasking purposes, such as modifications to the network sampling rate. The selected sampling rate for both sensor measurement and packet dissemination was 0.1 Hz, since the temperature and oxygen in the silage stack varied slowly. For a detailed description of the sensor unit the reader is referred to [8]. In order to protect the sensor node from damage during the ensiling, storage, and feed-out processes, a protective housing was developed (Figure 2) providing a maximum tolerance load of 15 kN for the weak axis and 32 kN on the strong axis of the sensor unit, which corresponds to a maximum tractor axle load of 43 kN. The experimental results showed that 98.9% to 99.3% of the packets disseminated from the tested sensor nodes were successfully delivered to the gateway. To transmit a packet including the temperature and humidity readings, approximately 12 mA is in total required. The power source is a 3.6 V, 1.2 Ah lithium battery. For the transmission frequency of 0.1 Hz the operational battery life is around 120 days and consequently a typical 4-month storage period can be safely monitored. The results of that study indicated that the designed wireless sensor nodes can be used in specific applications such as the one proposed in this paper.

## 4. The Computer Fluid Dynamics (CFD) Prediction Model

The general-purposes CFD software Fluent^®^ (Fluent Europe Ltd., Sheffield, UK) is used in the central server unit for the simulation of the course of the air temperature and the oxygen content within the storage facility. Fluent^®^ code uses a finite volume numerical scheme to solve the equations of conservation for the different quantities of flow (*i.e.*, mass, momentum, energy, and water vapor concentration). First, the code performs the coupled analysis of the pressure and velocity fields and then continues with the others parameters, *i.e.*, temperature or water vapor concentration. The fermentation process of the biomass is simulated using a customized routine built for the determination of the parameters characterizing the specific type of biomass at hand. At the present stage of the system’s development, a number of domains of interest have been development for experimental purposes and are available as a library. These domains were generated and meshed using Gambit, the integrated pre-processor software of Fluent^®^.

The CFD technique numerically solved the Navier-Stokes equations and the mass and energy conservation equations. The three dimensional conservation equations describing the transport phenomena for steady flows in free convection are of the general form:

(1) $$\frac{\partial (U\Phi )}{\partial x}+\frac{\partial (V\Phi )}{\partial y}+\frac{\partial (W\Phi )}{\partial z}=\Gamma \nabla^{2}\Phi +S_{\Phi }$$

In Equation (1), *Φ* stands for the transport quantity in a dimensionless form, while *U*, *V*, and *W* are the components of the velocity vector; *Γ* is the diffusion coefficient; and *S*_Φ_ is the source term.

The present flow and transport phenomena are described by the Navier-Stokes equations [26,27]. The standard k-ɛ model [28] assuming isotropic turbulence was adopted to describe the turbulent transport. The complete set of equations of the k-ɛ model can be found in [29]. The thermal buoyancy effect is approached through the Boussinesq model [27] which offers faster convergence, than considering the density variable in all equations. In this model the density is a constant value in all solved equations except from the buoyancy term calculation in the momentum equation. In this way the density is eliminated from the buoyancy term using the Boussinesq approximation. CFD enforces these conservation laws over a discretised flow domain in order to compute the systematic changes in mass, momentum and energy as fluid crosses the boundaries of each discrete region [30].

### 4.1. Biomass Modeling

The biomass was described using the equivalent macro-porous medium approach, which refers to the combination of the porous medium approach with a macro-model of heat and mass transfer between the forage and the surrounding air. Details for the porous media modeling can be found in [11].

Regarding the fermentation process a separate biochemical model was used. Oxygen diffusion is assumed to be unidirectionally downward from the top surface to the impermeable bottom, while dry matter is assumed to be reduced as a function of the respiration rate, the concentration of oxygen, and the concentration of substrate. The two following equations represent the mathematical model:

(2) $$\frac{\partial C_{OM}}{\partial t}=-k_{1}C_{O_{2}}C_{OM}$$

(3) $$\frac{\partial C_{O_{2}}}{\partial t}=D_{O_{2}}\frac{\partial^{2}C_{O_{2}}}{\partial x^{2}}-k_{2}C_{O_{2}}C_{OM}$$

where *C_OM_* is the concentration of organic matter (kg*_OM_* · m^−3^); *C*_*O*_2__ is the concentration of oxygen (O_2_/m^3^ of air); *t* is time (*h*); *k_1_* is the mass respiration rate (kg kg^−1^ h^−1^); *k_2_* is the oxygen depletion rate due to respiration m^3^ O_2_/m^3^ of air h^−1^); *D_O2_* is the oxygen diffusion rate (m^3^ h^−1^).

Respiration of organic matter in silage is assumed to follow the general chemical reaction of carbohydrate respiration estimated by the equation:

(4) $$k_{1}=0.9375\frac{m_{O_{2}}R_{a}}{C_{O_{2},0}}$$

where the constant 0.9375 is the mass ratio of depleted carbohydrates per unit oxygen, *m*_*O*_2__ is the mass density of oxygen (kg O_2_ m^−3^ O_2_), *R_a_* is the specific respiration rate of silage dry matter in air (m^3^ O_2_ kg^−1^ O_2_ h^−1^) and *C*_O_2_,0_ is the initial concentration of oxygen at *t* = 0 (0.21 m^3^ O_2_ m^−3^ gas)

The oxygen depletion rate is estimate as follows:

(5) $$k_{2}=\frac{R_{a}}{ɛC_{O_{2},0}}$$

where *ɛ* is the porosity (m^2^ O_2_ m^−3^ total volume). Porosity (ɛ) was calculated from the relationship [31]:

(6) ɛ=1−0.000919ρ

where *ρ* is the bulk density in *kg m^−3^*. Permeability (*y*) was calculated from the equation of [32]:

(7) y=673−0.614DM−0.619ρ

where DM is silage dry matter in g kg^−1^. The bulk density is the determined parameter that is connected with the type of the biomass. Each type of biomass is characterized by a specific bulk density which results to specific porosity and permeability values, affecting the physical processes related to the biomass storage.

A linear equation was used for the viscosity (*v*) of air as affected by temperature using values from [33,34]:

(8) $$v=(0.425\cdot T+19.84)\cdot 10^{6}.$$

### 4.2. Boundary Conditions

At the present state of the system’s development, a number of complete three dimensional (3D) models are available as a library. The selected control volume represents a large domain in order to simulate also the field where the monitored biomass storage facility is located. The cover was simulated as a solid zone composed by four rows of cells where the conduction thermal equation is being solved. A mixed heat transfer boundary condition (combination of radiation and convection with convective heat transfer coefficients) is applied at the external boundary of the solid region. Also, the same boundary condition is imposed at the internal margin where the solid and the fluid zones are coupled, restoring a conjugated heat transfer treatment at the specific area.

The grid was selected following grid independence test studies in order to ensure the solution independency from numerical errors due to spatial discretization. Grid quality was checked against the Fluent^®^ EquiAngleSkew (QEAS) criterion. At the inlet of the computational domain, a logarithmic inlet velocity profile (atmospheric boundary layer model) was considered [35]. The air velocity (at a reference height) and the air temperature are given as known values using the values of the meteorological mast.

### 4.3. Model Validation

A series of validation tests of the CFD model performance, in terms of the temperature and oxygen concentration predictions has been carried out. The tests concerned the comparison between the actual values (as the measured by the network sensors) and the ones predicted by the model (fitted with the updated actual weather conditions). The tests covered the whole time periods of sealing procedures, *i.e.*, from initiating sealing until the time when the oxygen concentration reached zero. Furthermore, special attention was paid to ensure safe storage conditions (*i.e.*, no damaged covers) since these are the precise conditions that the CFD has to emulate and simulate within the proposed diagnostic system.

**Figure 3.**
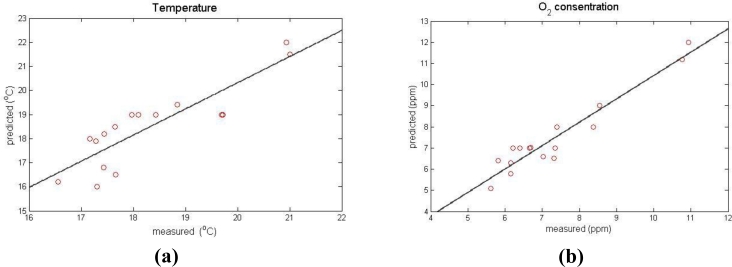
The correlation between measured (sensors network) and predicted (CFD model) values of the mean air temperature **(a)** and oxygen concentration **(b)**.

Figure 3 presents an instance of actual and predicted data (in a time step of 6 h). The error in the prediction of the air temperature (for all of the sensors) varied between 3 and 11%, with a correlation ratio of predicted (*T_pred_*) and measured values (*T_meas_*): *T_pred_* = 1.02 *T_mea_* and a correlation coefficient of 0.78. For the case of the oxygen concentration, the error in the prediction varied between 5 and 14%, with a correlation ratio of predicted (*O2_pred_*) and measured values (*O2_meas_*) as *O2_pred_* = 1.03 *O2_meas_* with a correlation coefficient of 0.90.

## 5. Case Study

In order to demonstrate the procedural functionality of the proposed system for monitoring and diagnosing interfering events within biomass storage facilities, a case study involving an experimental set-up and subsequent model estimations and evaluations was carried out and is presented.

### 5.1. Experimental Set-Up

**Figure 4.**
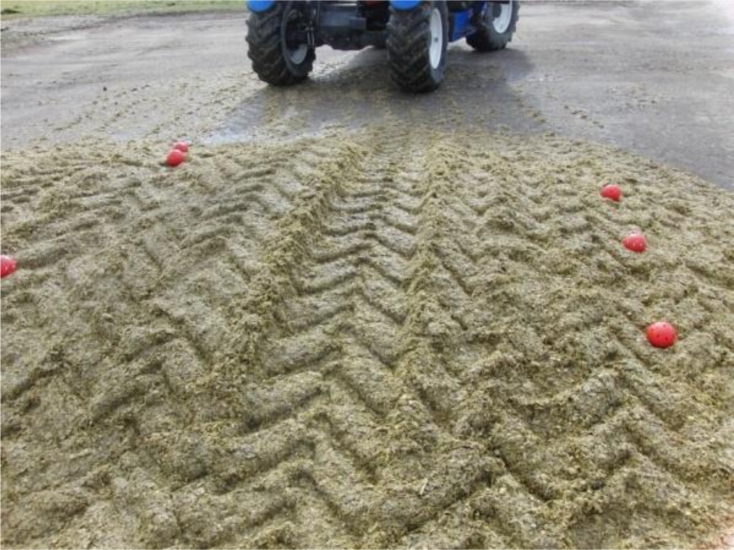
The sensor network within the prepared storage stack.

A semi-cylinder silage stack containing cut maize silage was used as a test storage facility. The dimensions of the silage stack were 3 m length, 1.5 m width, and 0.6 m height (Figure 4). The biomass, after its transportation from the field site to the storage site, was spread out in cumulative thin layers. Each layer was compacted (using an auxiliary tractor) and then the silage stack was sealed using an airtight cover as a way to prevent oxygen from entering the biomass material. The scope of the experiment was to examine the capability of the system to detect potential deviations in temperature and oxygen content values from the expected ones under the specific weather and storage conditions. The most usual reason for such a deviation is the presence of a tear in the plastic covering. To this end, the covering was, on purpose, penetrated generating a tear of 0.01 m diameter located in the right part of the centre of the stack. The experiment was carried out during May 2010.

**Figure 5.**
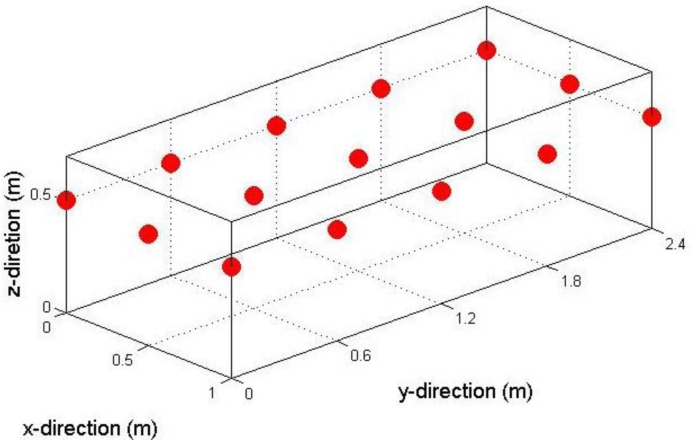
The configuration of the sensors network within the storage stack.

A network consisting of 15 sensor units was placed in a horizontal grid configuration at a distance of 0.10 m from the top of the silage stack (Figure 5). Measurement locations relatively close to the surface were selected because spoilage caused by the silage being exposed to air is initiated at near surface locations.

### 5.2. Model Calibration

The Fluent^®^ CFD code [36] was used as a basis where a required external source code for incident irradiance boundary conditions (written in C++) was embodied. The corresponding (Navier–Stokes) transport equations were solved numerically by a finite volume method, using a two dimensional structured mesh consisting of 16,000 cells. The final number of cells resulted from an empirical compromise between a dense grid, associated with a long computational time, and a less dense one, associated with a marked deterioration of the simulated results. Moreover, the grid quality was checked using the EquiAngleSkew criterion [36]. The EquiAngleSkew (QEAS) is a normalized measure of skewness. QEAS = 0 describes an equilateral element, and QEAS = 1 describes a completely degenerate (poorly shaped) element. In this study, the average value of QEAS was 0.1 in simulations indicated a high quality mesh [36]. The SIMPLEC [26] algorithm was used for pressure–velocity coupling, yielding an elliptic differential equation in order to formulate the mass conservation equation. The discretisation of the convective terms in the Reynolds averaged transport equations was materialized by the second order upwind scheme [37] and for the diffusive terms a central difference scheme was adopted. The convergence criterion of every time step was set to 10–7 for the continuity, momentum, and *k-ɛ* transport equations, while for energy and radiation equations the criterion was 10–8. The boundary conditions used for simulations were based on average values derived from the measurements provided by the meteorological station (*cf.* to the next section). The initial time interval for the present transient simulation was set to 1 s in order to ensure the appropriate small Courant-Friedrichs-Lewy factor (<0.5) although the defined 2nd order temporal discretization scheme is unconditionally stable. However, as the solution was proceed the time step was gradually increased. Finally, predicted numerical values were obtained in a frequency of six hours.

### 5.3. Weather Data

The weather data during the period of the experiment were obtained from the climate database developed and run by the Faculty of Agricultural Sciences at Aarhus University (Foulum Research Centre weather station [N6261335, E535275]). The updated weather data include hourly values of the following parameters: air temperature, air relative humidity, air wind speed, air wind direction, global solar radiation, surface temperature, and soil temperature in depths 0.1 m and 0.3 m.

### 5.4. Output / or Results / or Visualized Results

**Figure 6.**
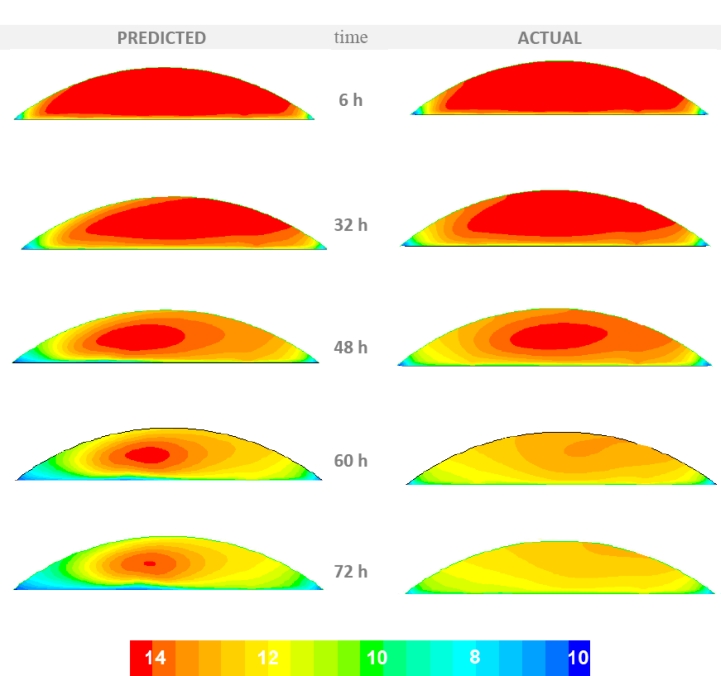
The predicted temporal distribution of the air temperature (at the stack center) by the CFD model (left) and the actual distribution according to the sensor data.

**Figure 7.**
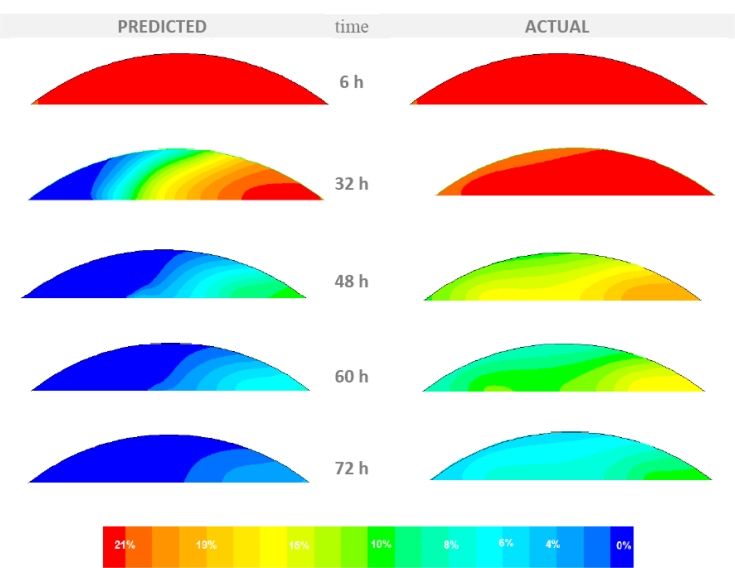
The predicted temporal distribution of the oxygen concentration (at the stack center) by the CFD model (left) and the actual according to the sensor data.

Figure 6 presents the predicted (by the CFD model) and the actual (as measured by the sensors) temporal distribution of the air temperature at a cross section perpendicular to the main axis of the storage stack located at its center. Figure 7 presents the predicted and actual oxygen content distribution at the same cross section. These figures are part of the visualization layer of the system that is simply a visual exception report. The purpose of this report is to deliver to the appropriate end-user the information of the possible occurrence of an unexpected event. The user can then evaluate this information and act if deemed necessary As it can be seen from the comparison of the two conditions (predicted and actual) the actual air temperature and oxygen content distributions inside the storage stack varied from the predicted ones. Specifically the actual values of temperature and oxygen content are considerably higher than the values predicted by the CFD model. This indicates that there is a high level of aerobic activity from microorganisms caused by a higher infiltration rate compared to the one under air-tight conditions. It can be deduced that probably there is damage in the storage covering allowing the infusion of outside air.

**Figure 8.**
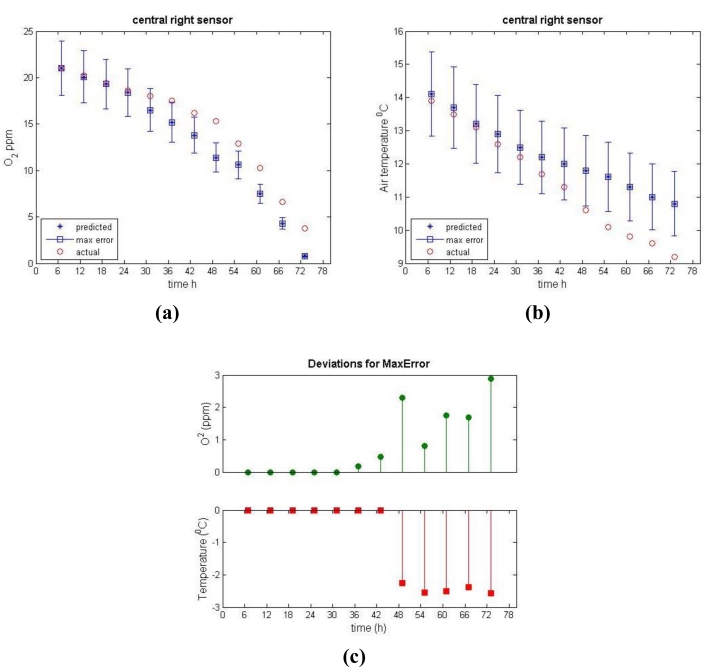
The course of the actual and predicted (with the maximum error incorporated) values of the oxygen concentration **(a)** and temperature **(b)**, and the deviations from the maximum error ranges **(c)**.

As a further functionality of the overall system the real differences between actual and predicted temperature and oxygen concentration must be identified. This implies integrating the measured difference and potential incorporated errors of the measurements. This integration is depicted in Figure 8, which provides the course of the actual value of the measured property (oxygen concentration in Figure 8(a) and temperature in Figure 8(b) combined with its predicted value where the maximum expected prediction error has been incorporated. The maximum prediction error was derived from the evaluation of the model and values that have been used in the presented case are: 11% for the case of the temperature and 14% for the case of the oxygen concentration.

As it can be seen from Figure 8 after the initial reduction of oxygen, the reduction rate was considerable higher in the predicted conditions. 36 h after the silage compaction there is the first indication for a real difference in the oxygen concentration. Specifically, the value measured by the sensor was 17.5%, which exceeds the error range of the predicted measurement which for the specific oxygen concentration predicted value of 15.2 is [13.1% 17.3%]. Based on this deviation, the 36 h defines the first alert from the system.

As regards the course of the air temperature, the first indication of a real difference appeared 48 h after air-tight covering. Specifically, the measured by the sensor value was 10.6 °C a value that exceeds the error range of the predicted air temperature which for the specific temperature value of 12 °C is [10.7 °C 13.3 °C]. At the 48 h time point both of the monitored properties are outside of the predicted values ranges, and this triggers a more definitive alert from the system.

## 6. Conclusions

This study has presented a framework for a diagnostic system capable of detecting potential changes in specific physicochemical properties, *i.e.*, temperature and oxygen content, during the biomass storage process. The diagnostic system comprises a monitoring tool based on a wireless sensor network and a prediction tool based on a validated computation fluid dynamics model.

Based on the results of the presented study, it was shown that the system can provide the manager (end-user) with information about the biomass quality parameters. The information can be provided to by graphical visualization as a first step and, as a second step, the system issues alerts depicting real deviations between actual and predicted values of the monitored properties.

The perspective of this diagnostic system is to be integrated in farm management information systems where the visualization and alerting can involve multi-delivery mechanisms such as instant messaging through e-mail, or wireless devices (e.g., cellular phones). This continuously updated information will provide the manager a solid basis for any necessary preventive measures.
